# Efficacy comparison of subcutaneous mastectomy using gasless and gas-insufflation single-port transaxillary approaches for gynecomastia

**DOI:** 10.3389/fsurg.2025.1562190

**Published:** 2025-04-11

**Authors:** Yuqing Zhang, Huiling Wang, Jie He, Yaqin Wu, Rui Liu, Xiangyuqin Xiao, Zheng Zeng, Chaojie Zhang

**Affiliations:** Department of Breast and Thyroid Surgery, The First Affiliated Hospital of Hunan Normal University/Hunan Provincial People's Hospital, Changsha, Hunan, China

**Keywords:** gynecomastia, subcutaneous mastectomy, endoscopic, surgical efficacy, postoperative recovery

## Abstract

**Background:**

To evaluate the clinical efficacy of gasless and single-port gas-insufflation transaxillary approaches in subcutaneous mastectomy for treating patients with gynecomastia (GM).

**Methods:**

This study enrolled 46 patients with GM from May 2022 to October 2023. Twenty patients underwent subcutaneous mastectomy using the single-port gas-insufflation transaxillary approach (gas-insufflation group), while the other 26 patients received the same procedure through the gasless transaxillary approach (gasless group). This study further conducted inter-group comparisons in terms of the operation time, intraoperative bleeding, volume of postoperative drainage, timing of drainage tube removal, short-term postoperative complications, length of postoperative hospital stay, and medical costs.

**Results:**

All the 46 patients completed the operation successfully without conversion to open surgery, with confirmed diagnosis of GM through pathology. The average surgical time for the gasless group was significantly shorter than that of the gas-insufflation group (38.20 ± 10.773 vs. 62.96 ± 15.311 min, *P* *<* *0.01*). There were no significant differences between groups in incision length, intraoperative bleeding, unilateral postoperative drainage volume, drainage tube retention time, length of postoperative hospital stay, or postoperative cosmetic outcomes (all *P* *>* *0.05*).

**Conclusion:**

This study supports the clinical feasibility of using gasless transaxillary approach for subcutaneous mastectomy of patients with GM.

## 1 Introduction

Gynecomastia (GM) is a common benign proliferation of the glandular tissue of the breast in men. Its prevalence is reported to be 32%–65%, depending on the age and the criteria used for definition (1). It arises from both physiological and non-physiological factors, which is characterized by unique clinical, histological, and radiological features. Physiological GM frequently occurs in neonates, adolescents, and older men, affecting up to 70% of the adolescent males (2). While non-physiological causes include chronic diseases such as cirrhosis, hypogonadism, renal insufficiency, as well as drug use, and rare tumors (3). Indeed, GM is often self-limiting and typically requires no specific intervention for asymptomatic cases. However, treatment may be necessary if symptoms persist beyond one year and induce severe pain, tenderness, or psychological distress (4). Medication and surgery are major therapeutic options in the clinical setting. While medication tends to be ineffective for patients with the conditions lasting over 12 months, thus necessitating surgical intervention (5). Currently, surgical treatments for GM include inframammary fold incision, parareolar incision adenomectomy, traditional open surgery, endoscopic-assisted adenomectomy, adenomectomy combined with liposuction, and Mammotome minimally invasive rotational excision. Each of these methods varies in indications, surgical trauma, postoperative recovery, and aesthetic outcomes (6–10). Recently, laparoscopic surgery through gasless axillary approach has gained popularity in thyroidectomy due to its safety and aesthetic outcomes (11, 12). However, there is no report concerning its use in treating GM. This study aims to evaluate the effectiveness of gasless single-port transaxillary and gas-insufflation single-port transaxillary approach for subcutaneous mastectomy in treating GM patients.

## 2 Methods

### 2.1 Patients

This study recruited patients diagnosed with GM between May 2022 and October 2023. Based on their preferences, the enrolled patients were assigned to either the gas-insufflation group or the gasless group. All participants received standardized preoperative education from the same surgical team, which detailed the advantages and disadvantages of both surgical methods, potential surgical risks, and expected postoperative recovery. Subsequently, patients selected their preferred method based on personal considerations. Those who chose the gas-insufflation approach were typically more familiar with other endoscopic procedures and viewed the gas-insufflation technique as more established and widely used. In contrast, those opting for the gasless approach often cited concerns about potential intraoperative or postoperative discomfort related to CO2 insufflation, such as subcutaneous emphysema or hypercapnia. Additionally, some patients considered economic factors, as the gasless method does not require a multi-channel laparoscopic single-hole puncture device or soft instrument sheath, thus reducing costs.

The surgeons provided recommendations based on preoperative physical examination and imaging assessments to ensure each patient was a suitable candidate for their chosen technique. However, the final decision rested with the patients. This method ensured that both groups were comparable in terms of baseline characteristics and that patient preferences did not introduce significant bias in surgical outcomes.

All surgical procedures were performed by the same lead surgeon, assisted by a consistent surgical team. This consistency ensured uniformity in surgical techniques and minimized potential bias related to differences in surgeon experience. Furthermore, all procedures adhered strictly to standardized surgical protocols.

### 2.2 Inclusion and exclusion criteria

Inclusion criteria: (1) Preoperative ultrasound-confirmed diagnosis of GM; (2) The disease significantly impacts the patient's daily life and causes psychological distress, leading the patient to actively seek surgical intervention; (3) Drug treatment is ineffective; (4) None of the patients had primary diseases such as hepatitis, endocrine disorders, or reproductive system abnormalities; (5) No underlying conditions such as hypertension, diabetes, cardiovascular diseases, or coagulation disorders.

Exclusion criteria: (1) Patients with gynecomastia persisting for less than one year; (2) Patients presenting with simple obesity; (3) Patients with secondary gynecomastia; (4) Patients diagnosed with breast cancer postoperatively; (5) Patients opting for medication as a treatment; (6) Patients with severe surgical contraindications, including cardiac, pulmonary, hepatic, or renal insufficiency.

### 2.3 Surgical steps

#### 2.3.1 Transaxillary approach, gasless group

Following successful general anesthesia, the patient was adjusted to a supine position with the operative side near the operating table's edge and the shoulders elevated. The arm on the operative side was abducted at 90°, aligning with the lower edge of the hand tray. A lipolytic solution of 400 ml was prepared, consisting of 200 ml saline, 200 ml Sterile Water for Injection (SWFI), 0.2 mg epinephrine hydrochloride injection, and 10 ml of 2% lidocaine. The use of SWFI reduces the osmotic pressure of the solution, creating a hypotonic environment that induces fat cells to absorb water, swell, and rupture, thus achieving effective lipolysis. The surgical field was routinely sterilized and covered with a sterile towel sheet. The surgical field was routinely sterilized, with the placement of a sterile towel sheet. Under the guidance of ultrasound, 100–150 ml of lipolytic solution was injected into the surface of the mammary glands and retromammary space. After 15 min, an incision of 4 cm in length was made along the transverse axillary line, about 4 cm from the axilla's top. Liposuction was performed in the retromammary space and on the subcutaneous glandular surface using a metal lateral orifice aspirator. A long-handled scalpel was utilized along the pectoral major muscle surface to dissociate the retromammary space, followed by the insertion of a luminal pull hook and luminal mirror. The ultrasonic scalpel and electrocoagulation hook were then used to release the retromammary space and subcutaneous glandular layer, aided by the luminal mirror. The nipple and areola were carefully dissociated using long-handled tissue scissors, with a small amount of glandular tissue retained at the nipple's back. After hemostasis and rinsing by saline, no significant bleeding was observed under the luminal microscope, followed by the placement of a drainage tube. The wound was sutured intermittently with a 3-0 absorbable suture for the subcutaneous tissue and dermis, and closed with a continuous intradermal tissue suture using a 4-0 absorbable suture. The procedure was completed with the application of an external negative pressure drainage bulb, sterile dressing, and pressure bandage.

#### 2.3.2 Single-port gas-insufflation group

After inducing general anesthesia, the patient was adjusted in the same supine position with the operative side close to the edge of the operating table and shoulders elevated. The upper limb on the operative side was abducted at 90° to lie flat against the hand tray. Similarly, a lipolytic solution of 400 ml (200 ml saline, 200 ml distilled water, 0.2 mg epinephrine hydrochloride, and 10 ml 2% lidocaine) was prepared prior to the surgery. The surgical field was sterilized, and a sterile towel was laid down. A curved incision of about 4 cm in length was made at the anterior axillary line and mammary gland edge intersection, where 150–200 ml of the prepared lipolytic solution was injected into the mammary gland's subcutaneous tissue and retromammary space, followed by a 15-min wait. A blunt dissection rod was used to separate the breast flap up to the breast margin marking line for the aspiration of the fat solution. A disposable multi-channel single-port laparoscopic trocar was inserted and carbon dioxide gas was introduced to maintain an 8–10 mmHg pressure to create an operating space. The nipple-areolar complex was suspended through suturing to secure more controlled and stable positioning, enhancing the surgical field and facilitating glandular dissection. The breast tissue was separated from the pectoralis major muscle using an electrocoagulation hook, ultrasonic scalpel, and dissecting scissors to fully separate and excise the breast glandular tissue and some fat tissue. The specimen was placed in a bag and removed through the port. A drainage tube was placed when no significant oozing detected under the luminal microscope after achieving hemostasis and rinsing using saline. The wound was sutured with a 3-0 absorbable suture intermittently for the subcutaneous tissue and dermis, and closed using a continuous intradermal tissue suture with a 4-0 absorbable suture. The surgery ended with the placement of an external negative pressure drainage bulb, sterile dressing, and pressure bandage.

### 2.4 Variables

The demographic characteristics of the two patient groups, including age, height, weight, and body mass index (BMI), were compared. The perioperative outcomes of both groups were systematically analyzed. Intraoperative data included operation time and intraoperative bleeding. Postoperative data encompassed the volume of drainage, timing of drainage tube removal, short-term complications, duration of hospital stay, and medical costs. Long-term follow-up data included patient satisfaction surveys.

### 2.5 Questionnaire

A simple questionnaire was developed to assess postoperative satisfaction of the recruited patients. This study employed the Visual Analog Scoring (VAS), using a 10 cm straight line to represent scores from 0 to 10. Patients marked points on the line based on their postoperative satisfaction. These marks were then quantified into scores, where a higher score indicated greater satisfaction of the patient (13).

### 2.6 Data analysis

SPSS 27.0 software was used to analyze the data statistically. Measurement data expressed as *x¯* ± *s* were analyzed by t-test, and counting data were analyzed by *χ*^2^ test or Fisher's exact probability test. The difference was regarded as statistically significant at *P* *<* *0.05*.

## 3 Results

There was no significant difference in age, height, weight, and body mass index (BMI) between the gasless group and gas-insufflation group (*p* *>* *0.05*) (Table 1). Furthermore, the gasless group had shorter operation time than that of the gas-insufflation group (*P* *<* *0.05*). There was no significant difference between the two groups in terms of incision length and intraoperative blood loss (*P* *>* *0.05*). Besides, no significant difference was observed in the comparison of unilateral postoperative drainage volume (ml), duration of drainage tube retention (d), length of hospital stay (d), and postoperative cosmetic satisfaction (points) between the two groups (*p* *>* *0.05*) (Table 2).

**Table 1:** Comparison of general conditions between groups.

Parameter	Gasless group (*n* = 20)	Gas-insufflation group (*n* = 26)	*t*	*p*-value
Age (years)	31.90 ± 17.31	31.54 ± 16.98	0.071	0.944
Height (cm)	171.12 ± 4.93	170.69 ± 6.67	0.257	0.351
Weight (kg)	72.49 ± 14.19	72.81 ± 11.40	−0.086	0.428
BMI	24.71 ± 4.61	25.02 ± 3.86	−0.247	0.513

**Table 2:** Comparison of operation methods, operation-related indices, perioperative complications, and short-term postoperative complications between groups.

Parameter	Gasless group (*n* = 20)	Gas-insufflation group (*n* = 26)	*t*	*p*-value
Operative time (min)	38.20 ± 10.77	62.96 ± 15.31	−6.149	<0.01
Incision length (cm)	4.25 ± 0.72	4.08 ± 0.56	0.891	0.379
Intraoperative blood loss (ml)	16.50 ± 4.32	14.81 ± 4.79	1.238	0.222
Unilateral postoperative drainage volume (ml)	47.70 ± 11.63	44.00 ± 12.54	1.023	0.312
Duration of drainage tube retention (d)	2.25 ± 0.44	2.35 ± 0.49	−0.691	0.493
Length of hospital stay (d)	2.70 ± 0.47	2.69 ± 0.47	0.055	0.956
Postoperative cosmetic satisfaction (points)	8.85 ± 0.671	8.77 ± 0.587	0.435	0.666

## 4 Discussion

GM is usually asymptomatic, although some patients may experience pain and tenderness in the breast (14). Simon et al. classified GM into four categories based on breast size and skin redundancy: I - small visible breast enlargement without skin redundancy; IIa - moderate breast enlargement without skin redundancy; IIb - moderate breast enlargement with skin redundancy; and III - marked breast enlargement with skin redundancy resembling a pendulous female breast, typically in obese patients (15). GM primarily arises from an imbalance between estrogen and androgen levels, which is idiopathic and physiologic in over 95% of cases (16). In adolescents, 75%–90% of GM cases are self-limiting and resolve within 1–3 years, with no treatment required in most cases. While patients with severe GM or those experiencing significant psychological distress should be treated and may benefit from pharmacological and surgical interventions. Medical treatments aim to correct the hormonal imbalance, which, however, is usually ineffective and may induce side effects. Surgical intervention is usually recommended for patients who need treatment. Meanwhile, as for pubertal GM, surgical management should be considered for nonobese male adolescents with persistent breast enlargement after a minimum 12-month period of observation, intractable pain, or significant psychosocial distress. In addition, surgical plans can be developed by referring to various classification systems based on clinical features (4, 17).

**Figure 1:**
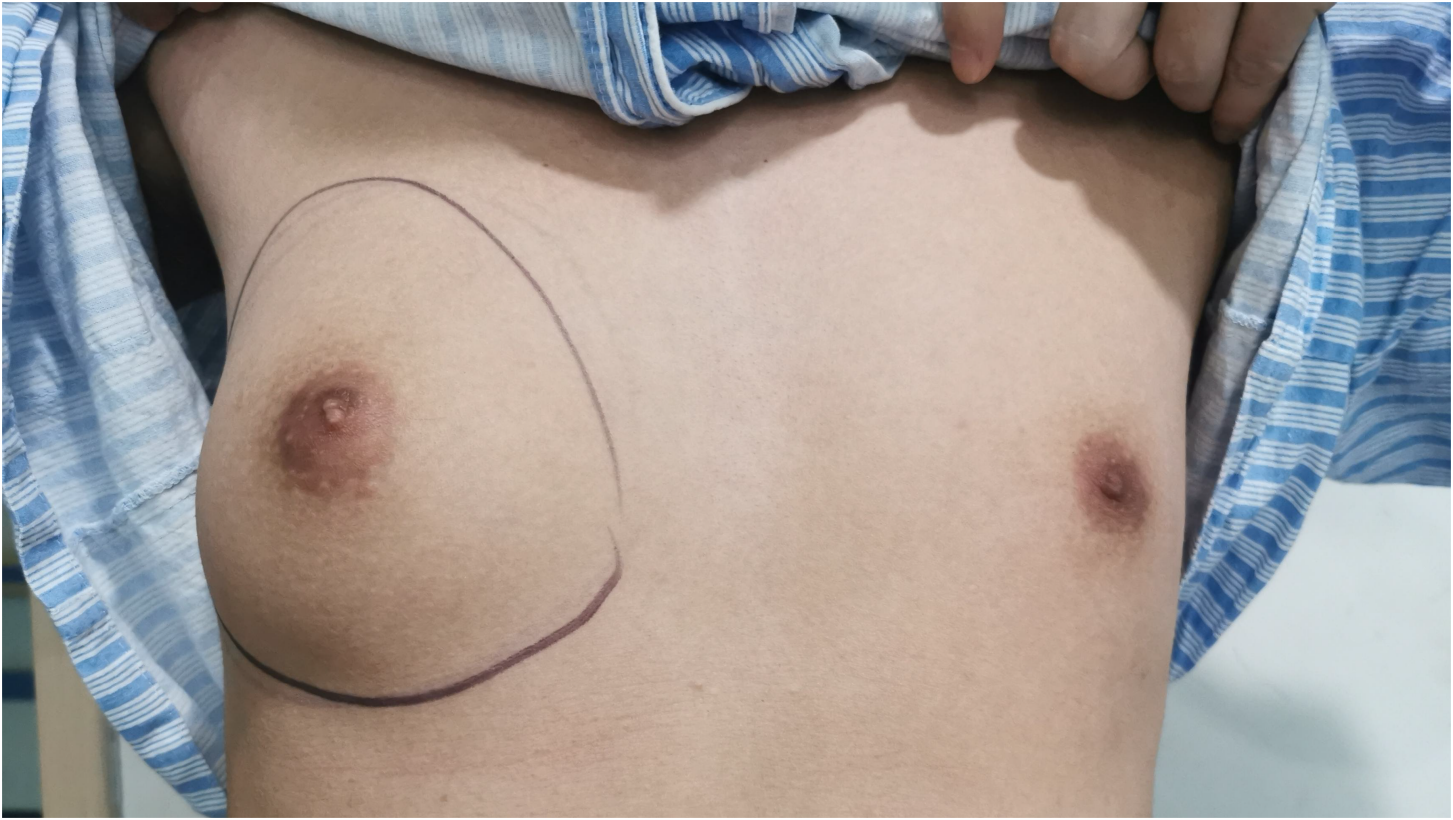
The patient's GM was classified as Simon grade IIa, with a duration of over one year.

**Figure 2:**
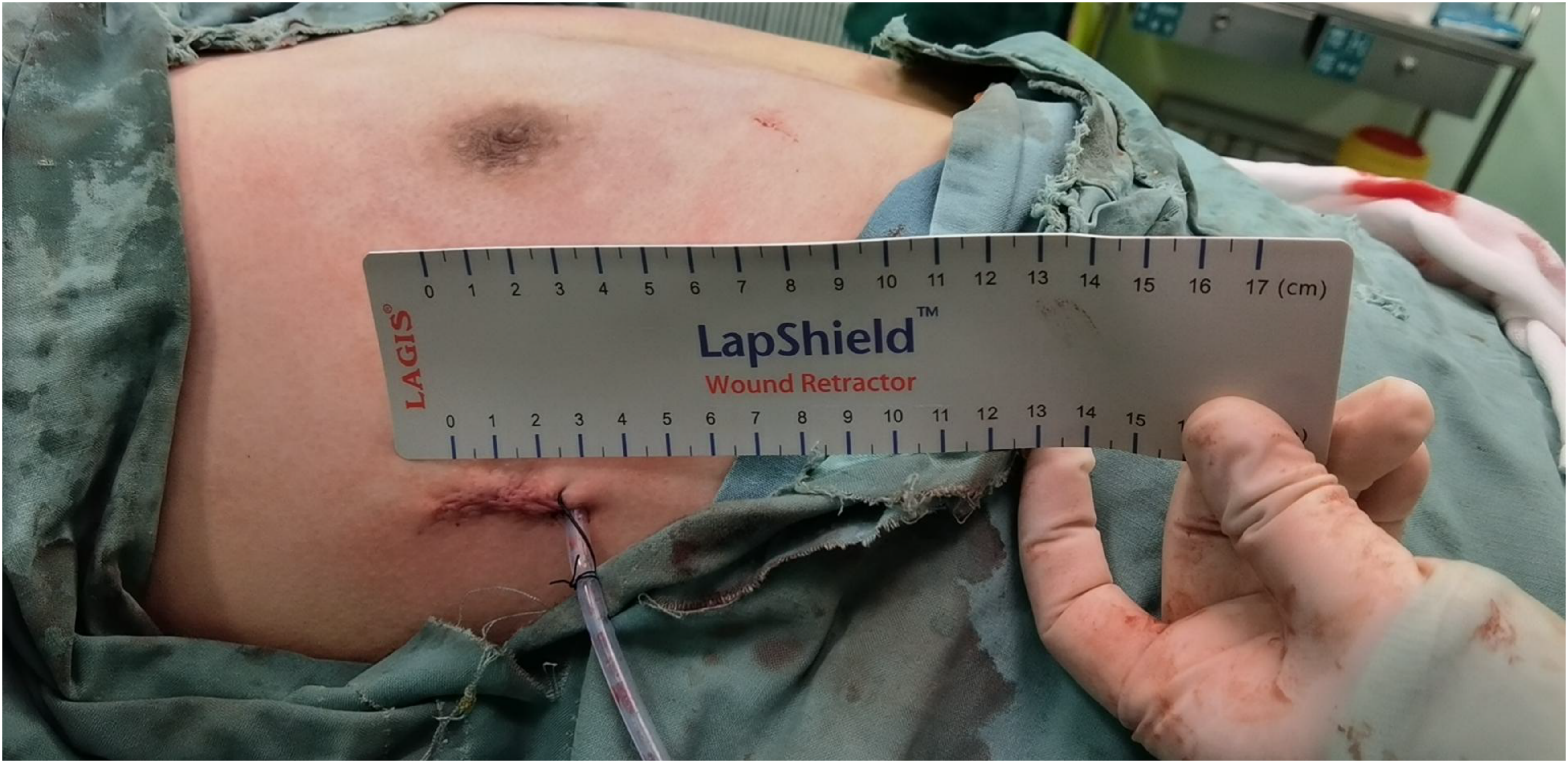
The effect after the patient underwent subcutaneous mastectomy through gasless single-port transaxillary approaches.

**Figure 3:**
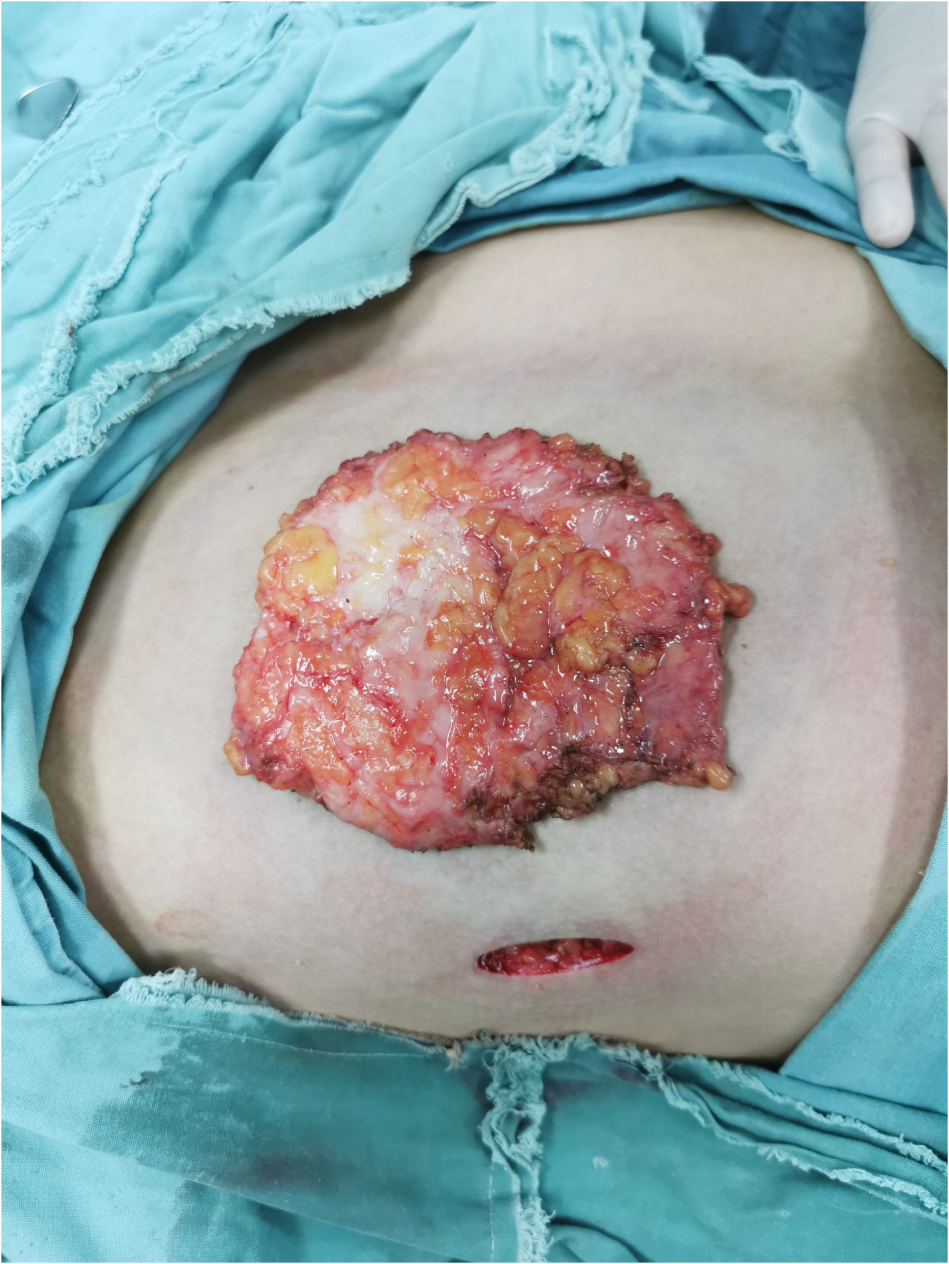
Complete excision of glandular tissue through subcutaneous mastectomy using gasless single-port transaxillary approaches.

**Figure 4:**
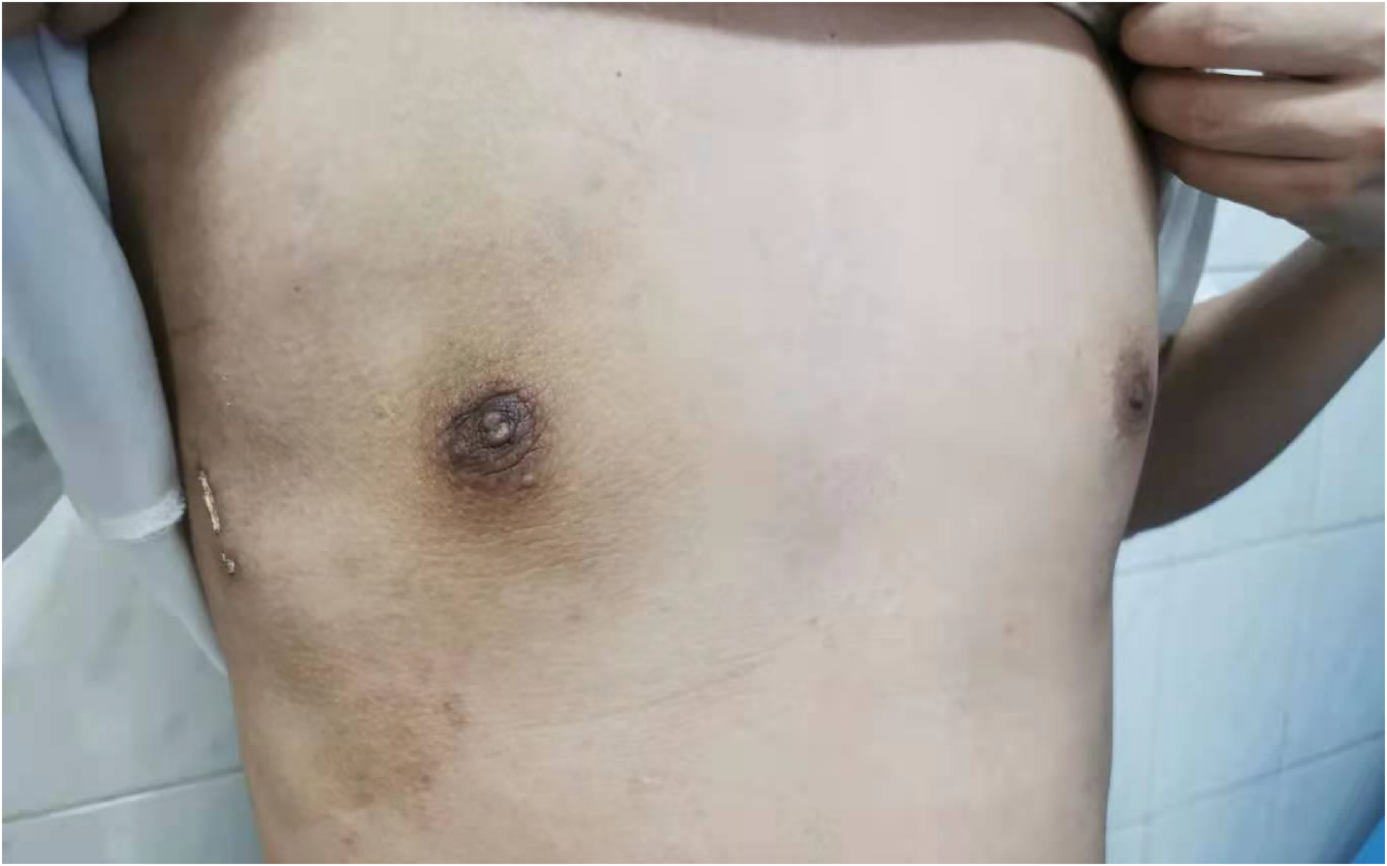
The recovery status of the patient a month after subcutaneous mastectomy through gasless single-port transaxillary approaches.

**Figure 5:**
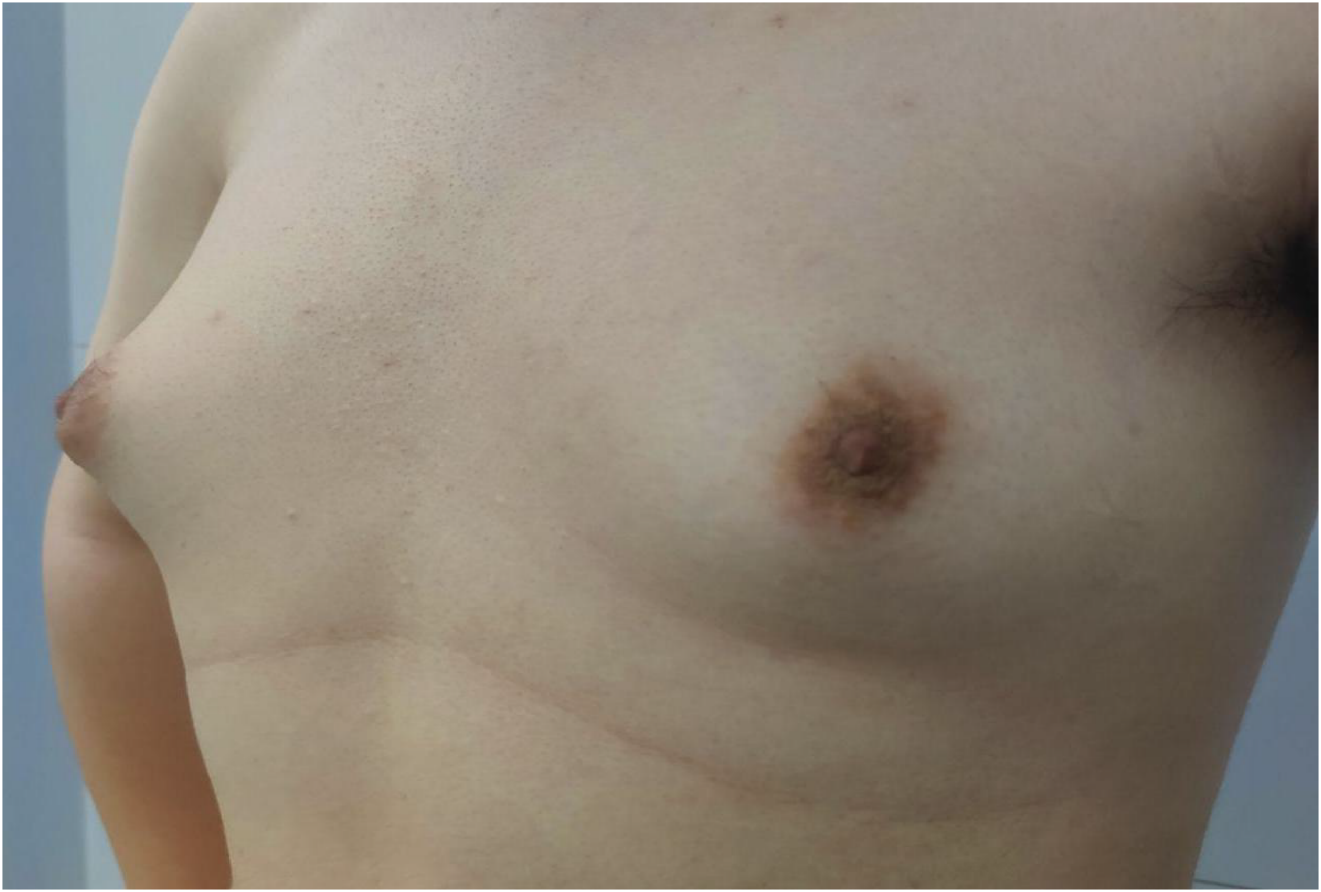
The patient's GM was classified as Simon grade IIa, with a duration of over one year.

**Figure 6:**
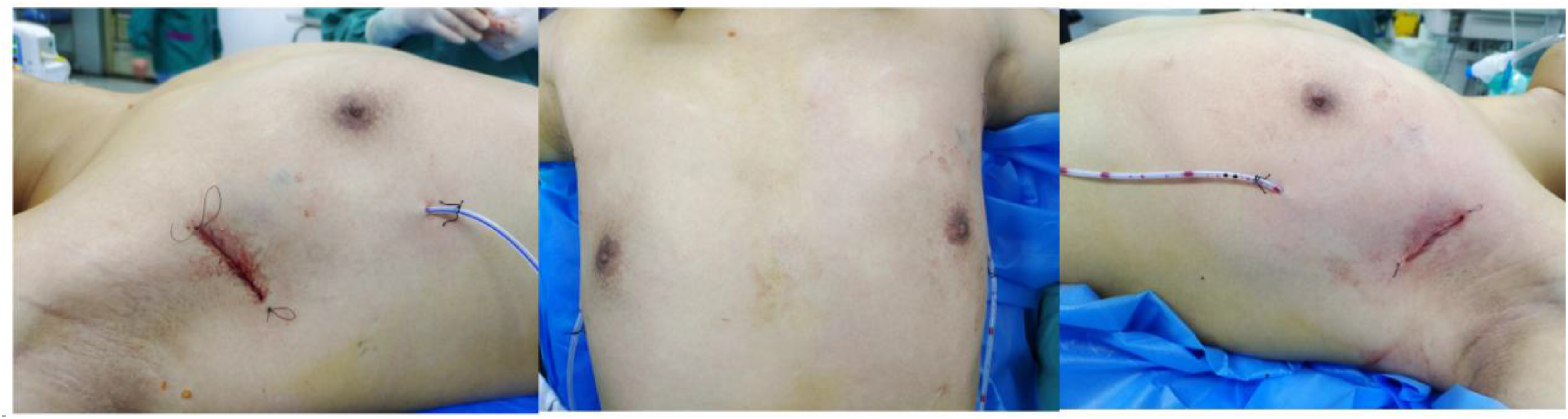
The effect after the patient underwent subcutaneous mastectomy through the single-port gas-insufflation transaxillary approach.

**Figure 7:**
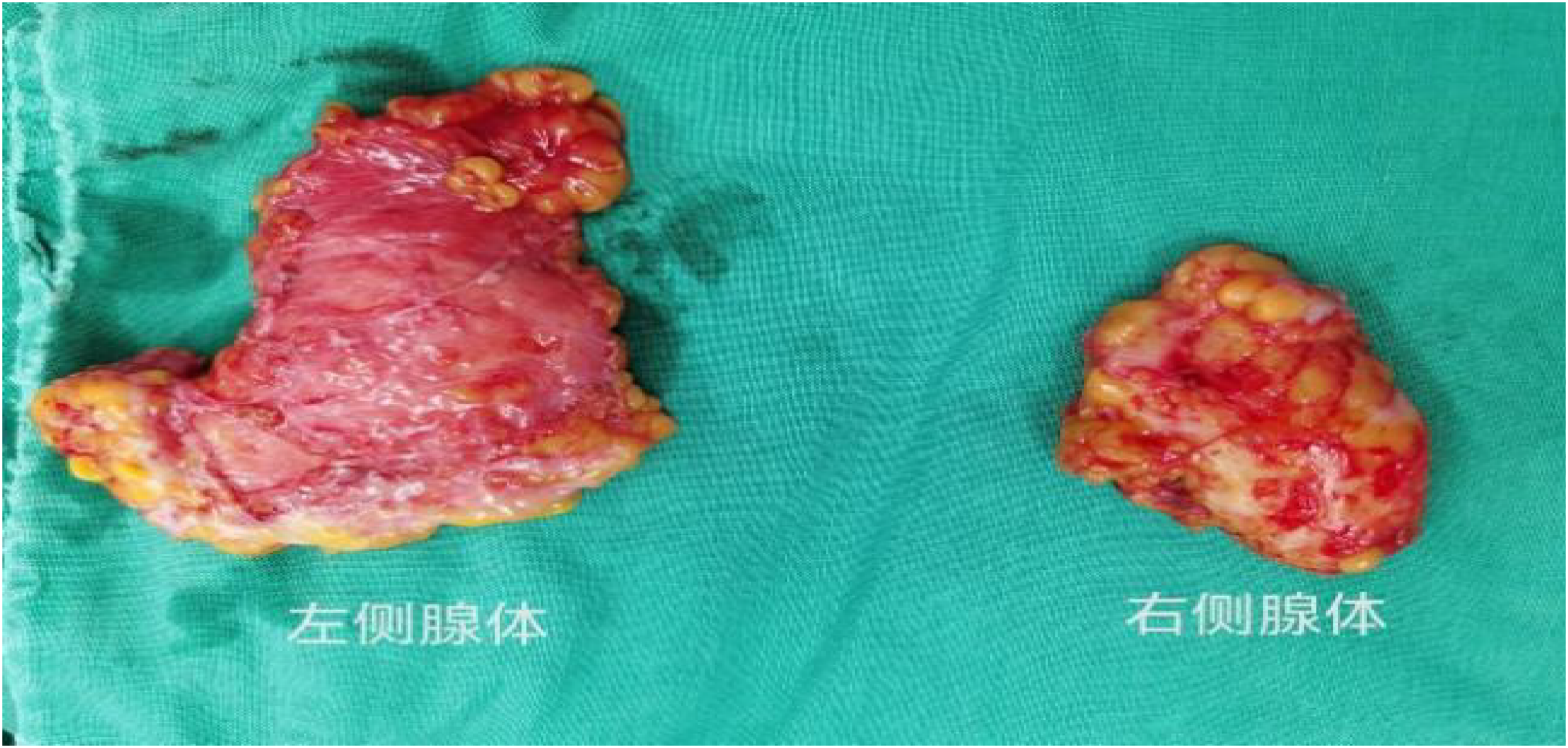
Complete excision of glandular tissue through subcutaneous mastectomy using the single-port gas-insufflation transaxillary approach.

**Figure 8:**
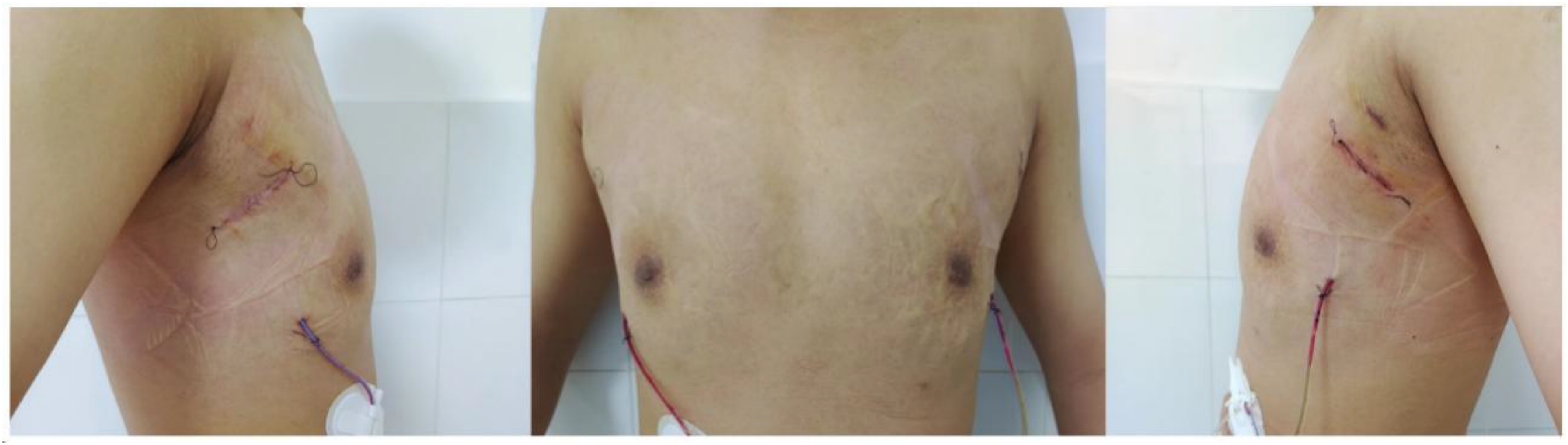
The day after surgery.

**Figure 9:**
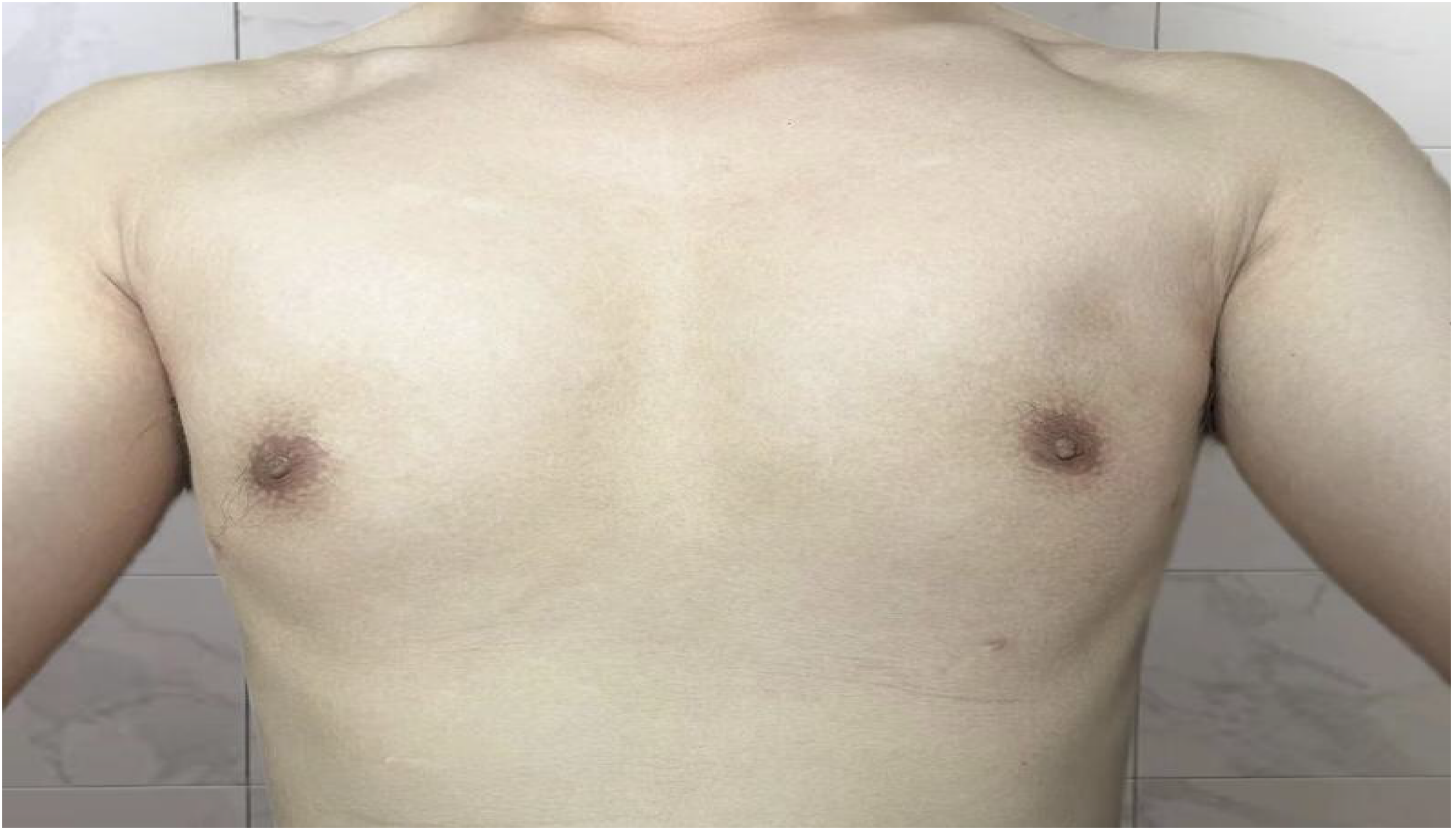
The recovery status of the patient three months after subcutaneous mastectomy through the single-port gas-insufflation transaxillary approach.

Considering the existing surgical options, traditional subcutaneous mastectomy via an inframammary approach is a traditional strategy allowing for complete gland excision, yet accompanied by significant scarring and emotional distress. Transareolar excision can provide more concealed wounds, but is unsuitable for large or deep lumps far from the areola (18). This method has the highest reported rate of nipple-areola complex (NAC) necrosis (18.1%) (19). Mammotome-assisted minimally invasive resection (MAMIR) is a new technique for treating GM. Despite no drainage tubes required, it may increase the risk of edema, bruising, and ischemic necrosis in the nipple-areola region if improperly performed (1, 20). It is not recommended for patients with breast masses larger than 6 cm in diameter or those located close to the skin surface or directly beneath the areola (2). For patients with grade 3 GM, redundant skin may require correction, but MAMIR is not effective in correcting skin sagging (21). Another method is endoscope-assisted subcutaneous mastectomy, which can be performed with or without CO2 insufflation. The approach to surgery is categorized into single-hole and triple-hole inflatable methods. Both techniques in this study employed a single-port endoscopic approach via the transaxillary route, avoiding the chest wall scars typical of open surgery. Figures 1–4 illustrate the preoperative appearance, intraoperative procedures, and postoperative recovery of patients treated with the gasless single-port transaxillary approach. Figures 5–9 depict the corresponding surgical and recovery outcomes of patients who underwent the single-port gas-insufflation transaxillary approach. This method is devoid of the chest wall poke holes seen in triple-hole lumpectomies, leaving the only scar concealed within the axillary cavity, hidden by the upper arm (Figures 2, 4, 6, 8). At the 3-month follow-up, no scar was visible on the front side of the patients (Figure 9), thereby maximizing the cosmetic outcome. This technique obviates the need for a periareolar incision and preserves the blood supply to the NAC, thereby mitigating the risk of postoperative ischemic complications. Meanwhile, this technique also enables complete excision of glandular tissue, minimizing the risk of residual lesions (Figures 3, 7). Additionally, it can also remove excess skin tissue to alleviate sagging caused by skin redundancy following surgery.

In this study, no significant difference was observed in surgical incision length, intraoperative bleeding volume, unilateral postoperative drainage volume, duration of drainage tube retention, length of hospital stay and postoperative beauty between the two group. However, patients undergoing subcutaneous mastectomy using the gasless transaxillary approach experienced shorter duration of operation than those through the single-port gas-insufflation transaxillary approach (*P* < 0.05); It can be explained by surgery via the gasless transaxillary approach can avoid the steps of inserting and inflating a single hole sleeve, and can also use open instruments. While endoscopic instruments are required when using the gas-insufflation method. The greater gripping force of open instruments as well as the relatively faster free and shear speeds can all reduce the difficulty of surgical operations and further accelerate the surgical speed. Moreover, no CO_2_ gas required to be injected during the surgical process when using gasless transaxillary approach, avoiding potential risks of CO_2_-insufflation-induced hypercapnia and gas embolism. Continuous negative pressure suction can maintain a clear surgical cavity without smoke, significantly reducing the risk of contamination of the surgical field of view and higher surgical safety accordingly (22); The use of a dedicated spatial construction system can maintain preferable and stable vertical and horizontal space, and also reduce labor costs (surgical assistant) (23). The auxiliary port broadens the operative field, enhances intraoperative visualization and flexibility, and facilitates precise dissection. During the procedure, tissues in the retroareolar region are dissected using a long-handled scalpel, circumventing the thermal spread resulted from electrothermal and ultrasonic devices, thereby mitigating the risk of thermal-induced skin necrosis. This technique emphasizes precise flap thickness control and careful handling of retroareolar tissues to preserve vascular integrity (24). Furthermore, no disposable multi-channel laparoscopic single-hole puncture device or soft instrument sheath is required when applying the gasless technique. It may result in a cost reduction of around 500–3,000 yuan compared to the gas-insufflation method, thereby reducing the economic burden on patients while preserving postoperative aesthetic outcomes.

In recent years, our department has used subcutaneous mastectomy through the gasless single-port transaxillary approach to treat GM with good results.

However, this study still has several limitations such as a short follow-up period and small sample size. We will continue to conduct in-depth evaluation of the safety, efficacy, and cosmetic outcome of subcutaneous mastectomy through the gasless single-port transaxillary approach for GM based on extended follow-up period and expanded sample size in the future.

## 5 Conclusions

In conclusion, subcutaneous mastectomy through the gasless single-port transaxillary approach for treating GM can minimize the operation time and cost, proving to be an effective and safe surgical option that can satisfy patients' cosmetic needs. Findings in this study supports the promotion of this method in the clinical setting under the prerequisite of strict adherence to surgical indications.

## Data Availability

The raw data supporting the conclusions of this article will be made available by the authors, without undue reservation.
